# *Pseudomonas chlororaphis* IRHB3 assemblies beneficial microbes and activates JA-mediated resistance to promote nutrient utilization and inhibit pathogen attack

**DOI:** 10.3389/fmicb.2024.1328863

**Published:** 2024-02-05

**Authors:** Dengqin Wei, Dan Zhu, Yunfeng Zhang, Zheng Yang, Yu Hu, Chun Song, Wenyu Yang, Xiaoli Chang

**Affiliations:** College of Agronomy, Sichuan Engineering Research Center for Crop Strip Intercropping System, Sichuan Agricultural University, Chengdu, Sichuan, China

**Keywords:** *Pseudomonas chlororaphis*, *Fusarium* root rot, rhizosphere microbiome, induced systemic resistance (ISR), root nodules

## Abstract

**Introduction:**

The rhizosphere microbiome is critical to plant health and resistance. PGPR are well known as plant-beneficial bacteria and generally regulate nutrient utilization as well as plant responses to environmental stimuli. In our previous work, one typical PGPR strain, *Pseudomonas chlororaphis* IRHB3, isolated from the soybean rhizosphere, had positive impacts on soil-borne disease suppression and growth promotion in the greenhouse, but its biocontrol mechanism and application in the field are not unclear.

**Methods:**

In the current study, IRHB3 was introduced into field soil, and its effects on the local rhizosphere microbiome, disease resistance, and soybean growth were comprehensively analyzed through high-throughput sequencing and physiological and molecular methods.

**Results and discussion:**

We found that IRHB3 significantly increased the richness of the bacterial community but not the structure of the soybean rhizosphere. Functional bacteria related to phosphorus solubilization and nitrogen fixation, such as *Geobacter*, *Geomonas*, *Candidatus Solibacter*, *Occallatibacter*, and *Candidatus Koribacter*, were recruited in rich abundance by IRHB3 to the soybean rhizosphere as compared to those without IRHB3. In addition, the IRHB3 supplement obviously maintained the homeostasis of the rhizosphere microbiome that was disturbed by *F. oxysporum*, resulting in a lower disease index of root rot when compared with *F. oxysporum*. Furthermore, JA-mediated induced resistance was rapidly activated by IRHB3 following *PDF1.2* and *LOX2* expression, and meanwhile, a set of nodulation genes, *GmENOD40b*, *GmNIN-2b*, and *GmRIC1*, were also considerably induced by IRHB3 to improve nitrogen fixation ability and promote soybean yield, even when plants were infected by *F. oxysporum*. Thus, IRHB3 tends to synergistically interact with local rhizosphere microbes to promote host growth and induce host resistance in the field.

## 1 Introduction

Soybean [*Glycine max* (L.) Merri.] is one of the most significant oilseed crops and also the main source of plant proteins for human and animal health in the world (Liu et al., 2020). Soybean root rot is one type of soil-borne disease caused by *Fusarium* spp., *Rhizoctonia solani*, *Phytophthora* sp., etc. (Ajayi-Oyetunde and Bradley, 2017; Chang et al., 2018) and often results in root and stem rot, seedlings yellowing and dwarfing, root hypoplasia, and nodule reduction (Tyler, 2007; Lin et al., 2012). This disease is typically characterized by wide distribution, serious damage, and difficult control, and therefore it has become the major cropping obstacle to soybean production worldwide (Chang et al., 2018; Cruz et al., 2020; Xu and Wei, 2020). In the last decades, several methods, including disease-resistant varieties, agricultural practices, and chemical seed coating, have been widely applied in disease control, but the yield losses are still estimated at 10–30% annually (Casida and Durkin, 2017; Cao et al., 2018; Zhang et al., 2020). Recently, biological control has received more attention because of its characteristics of being environment-friendly, pollution-free, requiring less reliance on chemical products, and having no resistance problem (Abdelaziz et al., 2023). Currently, some valuable biocontrol microbial agents have been constantly explored in different environmental samples and applied to the control of soybean root rot (Chang et al., 2022; Xu et al., 2022). Especially some beneficial bacteria or fungi isolated from the *in situ* rhizosphere of host plants have obvious advantages for disease control efficiency and display good potential for exploration (Xu et al., 2022; Wei et al., 2023).

The rhizosphere microbiome plays a vital role in maintaining the homeostasis of the rhizosphere microenvironment and ensuring plant health (Eisenhauer et al., 2010; Berendsen et al., 2012). Some beneficial rhizosphere microbes are responsible for nutrient uptake and substance conversion, while others have certain functions in biofilm formation, environmental communication, microorganism recognition, etc. This kind of diverse function among the rhizosphere microbiome helps host plants well adapt to or cope with complicated environmental stimuli, directly or indirectly (Compant et al., 2019; Arif et al., 2020). To be worthwhile, some rhizosphere beneficial microbes like plant growth-promoting rhizobacteria (PGPR) and fungi (PGPF) often trend to improve the diversity and structure of the rhizosphere microbiome in a conductive way to plant growth and health (Lugtenberg and Kamilova, 2009; Santoyo, 2022). For example, pre-inoculation of PGPR *Bacillus velezensis* NJAU-Z9 in seedlings changed the rhizosphere microbial structure of pepper in the field and especially increased the relative abundance of beneficial bacterial genera, including *Pseudomonas*, *Rhizomicrobium*, *Streptomyces*, *Lysobacter*, and the fungal genera, including *Cladorrhinum*, *Cladosporium*, and *Aspergillus*, which are positively associated with yield increase (Zhang et al., 2019). Using *Bacillus amyloliquefaciens* B1408 isolated from cucumber rhizosphere soil to control cucumber *Fusarium* wilt also remarkably increased the relative abundance of rhizosphere microbes, including *Streptomyces*, *Rhizobium*, *Acidovorax*, *Rhodanobacter*, *Mesorhizobium*, *Asticcacaulis*, and *Rhizoscyphus*, which were negatively correlated with the disease index of *Fusarium* wilt (Han et al., 2019).

In addition to the above, when host plants suffer from pathogen invasion, on the one hand, some rhizosphere PGPR act as strong competitors for space and nutrients or produce antibiotics to directly suppress, antagonistize, or kill pathogens (Cao et al., 2018; Abdelaziz et al., 2023). On the other hand, they can also induce host resistance, typically as induced systemic resistance (ISR) (Pieterse et al., 2014), which is distinguished from systemic acquired resistance (SAR) triggered by plant pathogens (Durrant and Dong, 2004). Furthermore, induced resistance is often regulated by a network of interconnected signaling pathways in which plant hormones play a major regulatory role (Pieterse et al., 2009). Generally, SAR is characterized by increased levels of salicylic acid (SA), which activates the expression of a large set of pathogenesis-related (PR) genes involved in defense responses (Durrant and Dong, 2004; Liu et al., 2016). In contrast, ISR is usually mediated by the jasmonic acid (JA) and ethylene (ET)-dependent pathways (Choudhary et al., 2007), which usually induce the expression of *PDF*, *LOX*, *PAL*, *ERF*, etc. (Bukhat et al., 2020; Abdelaziz et al., 2023). In some specific cases, ISR and SAR can share some components (Alizadeh et al., 2013). Some PGPG, like *Pseudomonas*, have been reported to induce host ISR in rice, grapes, tobacco, and *Arabidopsis*, significantly improve plant resistance, and effectively limit pathogen invasion (Pathak et al., 2004; Choudhary et al., 2007; Verhagen et al., 2010). In addition, the signaling pathways involved in the induction of ISR can be different depending on the microbial species and plant species (Alizadeh et al., 2013). So far, the mechanism of beneficial rhizosphere microbes-triggered ISR is still not clearly understood.

In general, the regulation mechanisms of PGPR in plant health are complex and diverse. In a previous study, we demonstrated that *Pseudomonas chlororaphis* IRHB3 had big potential for the control of soybean root rot and displayed good adaptation to environmental stresses through greenhouse experiments (Chang et al., 2022; Wei et al., 2023), but it is unclear about the biocontrol mechanism and efficacy of IRHB3 when applied in the field. Thus, we further introduced IRHB3 into the field soil to make it clear as follows: (i) whether IRHB3 as one kind of PGPR can play a role in promoting growth and disease resistance when applied in the field; and (ii) how IRHB3 regulates the rhizosphere microbiome and host resistance to impact soybean health.

## 2 Materials and methods

### 2.1 Field pot experiment design

Field soil was collected from a 5-year continuous cropping field in May 2022 at Qingpu Farm (103°88 N, 30°72 E). A 20-cm depth of farmland soil was shoveled up and mixed thoroughly. The soil had a pH of 5.02, a total N content of 1.99 g·kg^−1^, a total P content of 1.021 g·kg^−1^, an available N content of 254.68 mg·kg^−1^, and an available P content of 83.80 mg·kg^−1^, respectively.

Four treatments were designed in the field pot experiment, as follows: (i) Con, blank field soil; (ii) IRHB3, soil irrigated with biocontrol strain *Pseudomonas chlororaphis* IRHB3; (iii) Fo, soil inoculated by pathogenic *Fusarium oxysporum* B3S1 of soybean root rot; and (iv) IRHB3_Fo, soil co-treated with IRHB3 and B3S1. The cultivar Nandou12, moderately susceptible to *F. oxysporum*, was selected for pot experiments. Soybean seeds of Nandou12 were sequentially surface-disinfected with 30% H_2_O_2_ (V/V) for 10 min and washed three times with sterile water. Sterilized seeds were then transferred into a sterile budding box and cultured for 4 days at 28°C with 16 h of light and 8 h of darkness. Three healthy soybean seedlings with consistent growth were transplanted into plastic pots for four different treatments, as shown above.

For *F. oxysporum* inoculation (Fo), the pathogen *F. oxysproum* B3S1 was inoculated using the sorghum grain method as described by Chang et al. (2022). In short, sorghum grains were inoculated with B3S1 and cultured for 7–10 days at 25°C in the dark. Successfully infected sorghum was then dried at 25°C and ground into powder, and the abundance of B3S1 was approximately 2 × 10^7^ ppg per gram of sorghum powder. About 5 kg of field soil with 10% moisture content were mixed with 25 g of infected sorghum powder in one polypropylene pot (29 cm in diameter × 26 cm in height), which was covered about 6 cm above the un-infected soil layer above. Field soil without pathogen inoculation was used as the blank control (Con).

For IRHB3 treatment, the bacterium strain was inoculated through the bacterial suspension irrigation method. IRHB3 was cultured in LB liquid medium at 28°C, 150 rpm·min^−1^ for 24 h. After being centrifuged at 6,000 rpm·min^−1^ for 12 min, the bacterial pellets were collected and re-suspended using ddH_2_O to obtain the OD_600_ value of 0.5. About 1,000 mL aliquots of bacterial suspension were irrigated into each pot to ensure that per gram of soil contained approximately 107 cfu of IRHB3 as the treatment of IRHB3. The equal volume of IRHB3 bacterial suspension to irrigate the soybean seedlings grown in the *F. oxysporum*-inoculated soil was used as the treatment of IRHB3_Fo. Each treatment contained 15 replicative pots, and each pot contained three seedlings. All pots were placed in a randomized complete block design in the field. The detailed methods were described as shown in Supplementary Figure S1.

### 2.2 Sampling and analysis of roots and rhizosphere soils

Rhizosphere soil and plant roots from the four treatments (Con, IRHB3, Fo, and IRHB3_Fo) described above were sampled at the growth period (V5), flowering period (R2), and harvest period (R8) of soybeans, respectively. For plant sampling, a total of 15 plants per treatment were dug up during the corresponding period. After being watered with tap water, the disease symptom of root rot was observed, and the disease occurrence rate as well as the disease index (DI) were calculated as described by Chang et al. (2018). Plant roots were then scanned and analyzed using the LD-WinRHIZO Plant Root System Analyzer (RHIZO 2009, Quebec, Canada), and growth parameters, including the shoot dry weight, root dry weight, nodule fresh weight, and seed fresh weight per plant, were measured. For quantitative real-time PCR (qRT-PCR) analysis, parts of root tissues were randomly cut into small pieces, thoroughly mixed, frozen in liquid nitrogen for 30 min, and finally stored at −80°C.

For soil samples, rhizosphere soil was carefully collected by shaking off the soil blocks from the plants and then brushing the soil tightly attached to the root surface using a hairbrush. Samples from the same treatment were mixed, thoroughly homogenized, and then divided into two parts. One part of the rhizosphere soil was air-dried for the analysis of soil characteristics according to the method established by Xiong et al. (2015), whereas the other part was stored at −80°C for high-throughput sequencing analysis.

### 2.3 Genome DNA extraction and high-throughput sequencing of rhizosphere soil bacterial community

For each soil sample (20 in total: four treatments × five replicates), genomic DNA was extracted from 0.5 g of rhizosphere soil using the E.Z.N.A.^®^ Soil DNA Kit (Omega Bio-tek, Norcross, GA, USA) according to the manufacturer’s protocols. DNA concentration and purity were determined using a NanoDrop ND-2000 spectrophotometer (NanoDrop Technologies, Wilmington, DE, USA). The V1-V9 region of the bacteria *16S rRNA* gene was amplified using primers 27F and 1492R (Supplementary Table S1) in a 20- μL PCR reaction system, which contained 4 μL of 5× FastPfu Buffer, 2 μL of 2.5 mM dNTPs, 0.8 μL of each primer (5 μM), 0.4 μL of FastPfu Polymerase, and 10 ng of template DNA. PCR was performed at 95°C for 2 min, followed by 27 cycles at 95°C for 30 s, 55°C for 30 s, and 72°C for 60 s, and a final extension at 72°C for 5 min. Amplicons were examined by 2% agarose gels and purified using the AxyPrep DNA Gel Extraction Kit (Axygen Biosciences, Union, CA, USA) according to the manufacturer’s instructions.

The sequencing of purified SMRTbell libraries generated from the Zymo and HMP mock communities was conducted on dedicated PacBio Sequel II 8 M cells using the Sequencing Kit 2.0 chemistry, while purified SMRTbell libraries generated from the pooled and barcoded samples were sequenced on a single PacBio Sequel II cell. All amplicons were sequenced by Shanghai Biozeron Biotechnology Co. Ltd. (Shanghai, China). The SMRT Link Analysis software version 9.0 was employed to process PacBio raw reads to obtain demultiplexed circular consensus sequence (CCS) reads with the minimum number of passes = 3 and minimum predicted accuracy = 0.99, respectively. Raw reads were processed using the SMRT Portal to ensure sequence length (<800 or > 2,500 bp) and quality. After that, sequences containing 10 consecutive identical bases were further filtered to remove barcodes, primer sequences, primers, and other sequences. OTUs were clustered with 98.65% similarity cut-off using UPARSE 7.1,^1^ and chimeric sequences were identified and removed using UCHIME. The phylogenetic analysis of each *16S rRNA* gene sequence was conducted through the RDP Classifier^2^ against the silva (SSU132) *16S rRNA* database using a confidence threshold of 70% (Amato et al., 2013).

### 2.4 Bioinformatics analysis for high-throughput sequencing

The rarefaction analysis was performed using Mothur v.1.21.1 (Schloss et al., 2009) to disclose the diversity indices, including the Chao, Shannon, and Evenness diversity indices. The beta diversity analysis, including the values of PCOA and NMDS, was conducted through UniFrac (Lozupone et al., 2011). The Spearman’s correlation coefficients were assessed to determine the relationships between bacteria and soil physicochemical factors. Correlation was considered to be significant when the absolute value of Spearman’s rank correlation coefficient (Spearman’s *r*) was >0.6 and statistically significant at the value of *p* < 0.05. All statistical analysis was performed by the R *stats* package. The R (*pheatmap* package) and Cytoscape^3^ were used to evaluate the relationships through correlation heatmaps and network diagrams, respectively. Venn diagrams were prepared by Draw Venn Diagram^4^ to analyze overlapped and unique OTUs. One-way permutation analysis of variance (PERMANOVA) was conducted using the R vegan package to assess the statistically significant impacts of treatment processes on bacterial communities.

### 2.5 Quantification of *Pseudomonas chlororaphis* IRHB3 and *Fusarium oxysporum* B3S1 in rhizosphere soil samples

Quantitative real-time PCR (qRT-PCR) was used to determine the abundance of *P. chlororaphis* IRHB3 and *F. oxysporum* B3S1 in soybean rhizosphere soil from different treatments, and the specific fragments of *gryB* and *rDNA ITS* genes were amplified using primer pairs PC03F/PC03R and FOF/FOR1 (Jiménez-Fernández et al., 2010) for the quantification of IRHB3 and B3S1, respectively (Supplementary Table S1). Briefly, total DNA was extracted from 0.5 g of rhizosphere soil using the DNasy Power Soil Pro Kit (Qiagen, Stuttgart, Germany) according to the manufacturer’s protocols. Then, qRT-PCR was performed on the Chromo4 Real-Time PCR System (Bio-Rad, Hercules, CA, USA) in a 10-μL reaction mixture containing 5 μL of the SYBR qPCR Mix (2×) (Vazyme-Bio, Shanghai, China), 1 μL of each primer (10 mM), 1 μL of template DNA (10 ng), and 2 μL of ddH_2_O. The PCR thermal cycling conditions were set as follows: 3 min at 95°C for heat activation, 40 cycles of 10 s at 95°C, 30 s at annealing temperature (Supplementary Table S1) for amplification, 15 s at 95°C, 1 min at 60°C, and 15 s at 95°C for melting curve analysis. The *16S rRNA* for *P. chlororaphis* and the *ITS* for *F. oxysporum* were used as the reference genes. Gene expression values were calculated as Log10 values (target copy number per gram soil) prior to further statistical analysis. This experiment for each sample included four technical replicates and three biological replicates.

### 2.6 Expression analysis of defense and nodulation signaling genes in soybean root

The total RNA of soybean roots harvested from four treatments in the field was extracted using the Fast Pure^®^ Universal Plant Total RNA Isolation Kit (Vazyme-Bio, Chengdu, China). The RNA concentration was quantified using the NanoDrop fluorometer (Thermo Fisher Scientific, Stuttgart, Germany). The first-strand cDNA was synthesized using 2.5 μg of RNA in a 20-μL reaction volume according to the instructions of the BeyoRT^™^ II First Strand cDNA Synthesis Kit (Beyotime-Bio, China). Four genes, including *PR1*, *PR5*, *PDF1.2*, and *LOX2*, were selected to examine JA or SA signaling-related resistance (Lenis et al., 2010), whereas three genes, *GmENOD40b*, *GmNIN-2b*, and *GmRIC1*, were amplified to analyze the nodulation formation (Hayashi et al., 2012). Specific primer pairs used in this study are listed in Supplementary Table S1. qRT-PCR was performed in 10-μL reaction mixtures containing 5 μL of the SYBR Qpcr Mix (2×), 1 μL of 10 mM of each primer (Supplementary Table S1), 1 μL of cDNA templates (diluted five times from the original cDNA synthesis reaction), and 2 μL of ddH_2_O. The PCR thermal cycling conditions were the same as in Section 2.5. Gene relative expression was investigated using the 2^−ΔΔ Ct^ method (Livak and Schmittgen, 2001), with the *GmActin* gene as an endogenous reference for normalization. There were three biological replicates and four technical replicates.

### 2.7 Statistical analyses

High-throughput sequencing statistical analyses and data visualization were performed by R 4.3.1 (R software, Auckland, New Zealand). qRT-PCR raw data were analyzed using Excel 2010 (Microsoft Excel software, Washington, USA) by the 2^−ΔΔCt^ method. Differential analysis and visualization of soybean field agronomic traits, soil chemistry, and gene relative expression were conducted using SPSS 24 software (SPSS Software Inc., Chicago, IL, USA) and GraphPad Prism 10 (GraphPad Software Inc., San Diego, CA, USA), respectively. One-way analyses of variance (ANOVA) with the Duncan’s multiple range test were performed for multiple comparisons.

## 3 Results

### 3.1 IRHB3 promotes soybean growth and suppresses root rot in the field

**Figure 1:**
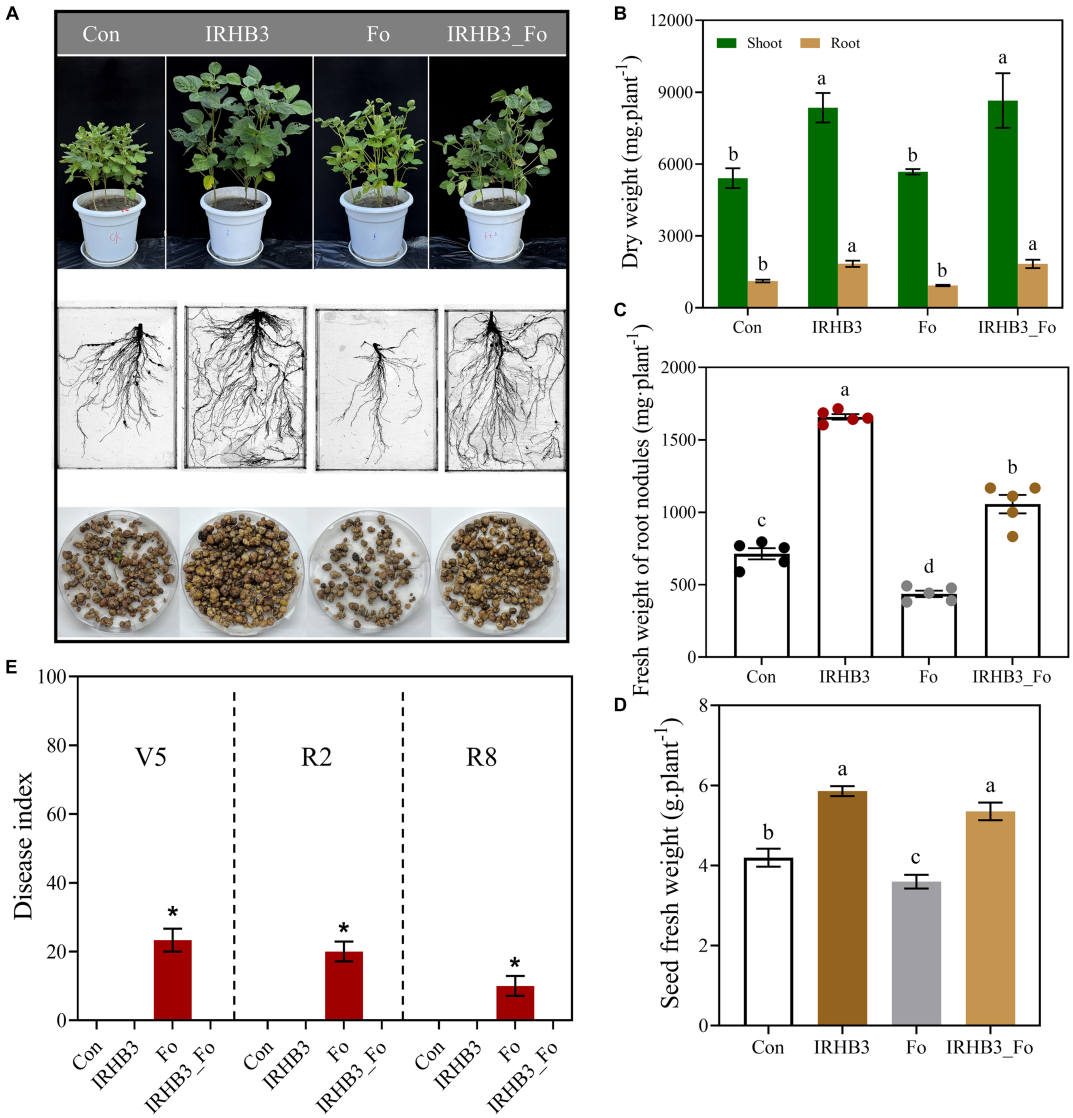
Effects of IRHB3 on soybean growth, *Fusarium* root rot, and soybean yield. **(A)** Effects of different treatments on shoot growth (upper), root development (middle), and nodule formation (lower) of soybeans at R2 period. **(B)** Dry weight of shoots and roots of soybeans at the R2 period. **(C)** Nodule fresh weight of soybean at R2 period. **(D)** Seeds fresh weight of soybean at R8 period. **(E)** Disease index of *Fusarium* root rot at V5, R2, and R8 periods. The data represent the mean ± SEM. Con, blank field soil; IRHB3, soil irrigated with biocontrol strain *Pseudomonas chlororaphis* IRHB3; Fo, soil inoculated by pathogenic *Fusarium oxysporum* B3S1 of soybean root rot; and IRHB3_Fo, soil co-treated with IRHB3 and B3S1. Asterisks or different lowercase letters indicate significant differences (*p* < 0.05) according to Duncan’s multiple range test, similarly hereinafter.

As shown in Figure 1A, soybean seedlings grown in the IRHB3-supplemented soils (IRHB3 and IRHB3_Fo) were the most robust, with the highest and strongest stems and lush green leaves at the R2 growth stage. Specifically, plant roots were obviously well developed after IRHB treatment (IRHB3), which were characterized by strong taproots, abundant lateral roots, and increased root nodules. Compared with those in the blank soil (Con), the dry weight of plant stems and roots after IRHB3 treatment was increased by 54.35 and 64.87%, respectively (Figure 1B), and meanwhile, the nodule fresh weight also sharply rose up by 132.55% and went up to 1658.96 mg per plant (Figure 1C). Furthermore, seed fresh weight reached 5.86 g per plant in the IRHB3-treated soil as compared to 4.20 g in the blank soil, which increased by 39.60% (Figure 1D). Interestingly, IRHB3 has similar effects on soybean growth at the R2 period to those at the V5 and R8 periods, and the detailed data are listed in Supplementary Figure S2.

To evaluate the IRHB3 impacts on root rot disease, we found that *F. oxysporum* inoculation (Fo) had negative impacts on plant growth and root development, whereas the IRHB3 supplement (IRHB3_Fo) largely alleviated these negative effects caused by *F. oxysporum* (Figure 1A). The dry weight of shoots and roots after IRHB3 addition reached 59.94 and 64.20%, respectively, which was remarkably higher than those in the treatments of *F. oxysporum* (Fo) and even control (Con) (Figure 1C). In addition, pathogen inoculation seriously decreased soybean nodule formation, and the abundance of root nodules was 38.83% less than those in the control (Con). Interestingly, the nodulation ability in the combined treatment of *F. oxysporum* and IRHB3 (IRHB3_Fo) was no longer restricted by pathogens, and instead, the nodule fresh weight significantly rose by 141.92 and 47.99% as compared to *F. oxysporum* and control, respectively. Meanwhile, seed fresh weight affected by *F. oxysporum* had also dramatically recovered and even increased by 27.52% as compared to control (Figure 1D). At all three growth periods, *F. oxyporum* inoculation caused the highest disease index and accounted for 23.33% (V5), 20% (R2), and 10% (R8), respectively (Figure 1E), whereas almost no root rot symptoms were observed in the combined treatments of IRHB3 and *F. oxysporum* (IRHB3_Fo). Thus, IRHB3 has positive effects on soybean growth and root rot suppression.

### 3.2 IRHB3 promotes soybean uptake of available nitrogen and phosphorus from soil

In order to evaluate the effects of four treatments on soil physicochemical properties, several parameters, including total phosphorus (TP), total nitrogen (TN), available phosphorus (AP), alkaline nitrogen (AN), and pH, were examined at the R2 stage. As shown in Table 1, three parameters, including TP, TN, and pH, of the rhizosphere soil were not affected by all treatments, but the contents of AP and AN were obviously distinct. The contents of AP varied from 84.19 to 8.65 mg·kg^−1^ with the trend of IRHB3 > Con > IRHB3_Fo > Fo. Compared with control, both treatments of IRHB3 and IRHB3_Fo increased AP by 5.31 mg·kg^−1^ and 5.45 mg·kg^−1^, respectively. The contents of AN ranged from 236.11 to 284.67 mg·kg^−1^ with the trend of IRHB3 > IRHB3_Fo > Con > Fo, and comparison of IRHB3 vs. Con and IRH3_Fo vs. Fo showed AN contents were increased by 41.316 mg·kg^−1^ and 32.23 mg·kg^−1^, respectively. Thus, IRHB3 has positive impacts on the increase of AN and AP, and also partially recovers the ability of AN and AP utilization.

**Table 1:** Rhizosphere soil physicochemical properties of different treatments examined in this study.

Treatment	TP (g·kg^−1^)	TN (g·kg^−1^)	AP (mg·kg^−1^)	AN (mg·kg^−1^)	pH
Con	1.09 ± 0.03a	1.90 ± 0.01a	93.33 ± 0.30b	243.36 ± 0.43c	5.04 ± 0.01a
IRHB3	1.04 ± 0.01a	1.95 ± 0.03a	98.65 ± 0.64a	284.67 ± 0.78a	5.05 ± 0.01a
Fo	1.04 ± 0.02a	1.89 ± 0.03ab	84.19 ± 0.03d	236.11 ± 0.99d	5.08 ± 0.01a
IRHB3 + Fo	1.04 ± 0.01a	1.94 ± 0.03a	89.64 ± 0.24c	268.35 ± 0.67b	5.07 ± 0.03a

TP, total phosphorus; TN, total nitrogen; AP, available phosphorus; AN, alkaline nitrogen. Data are means ± SEM. Different lowercase letters in the same row mean significant differences (*p* < 0.05) according to Duncan’s *multiple range* test, similarly hereinafter.

### 3.3 Dynamic tracking and correlation analysis of IRHB3 and *Fusarium oxysporum* in soybean rhizosphere

**Figure 2:**
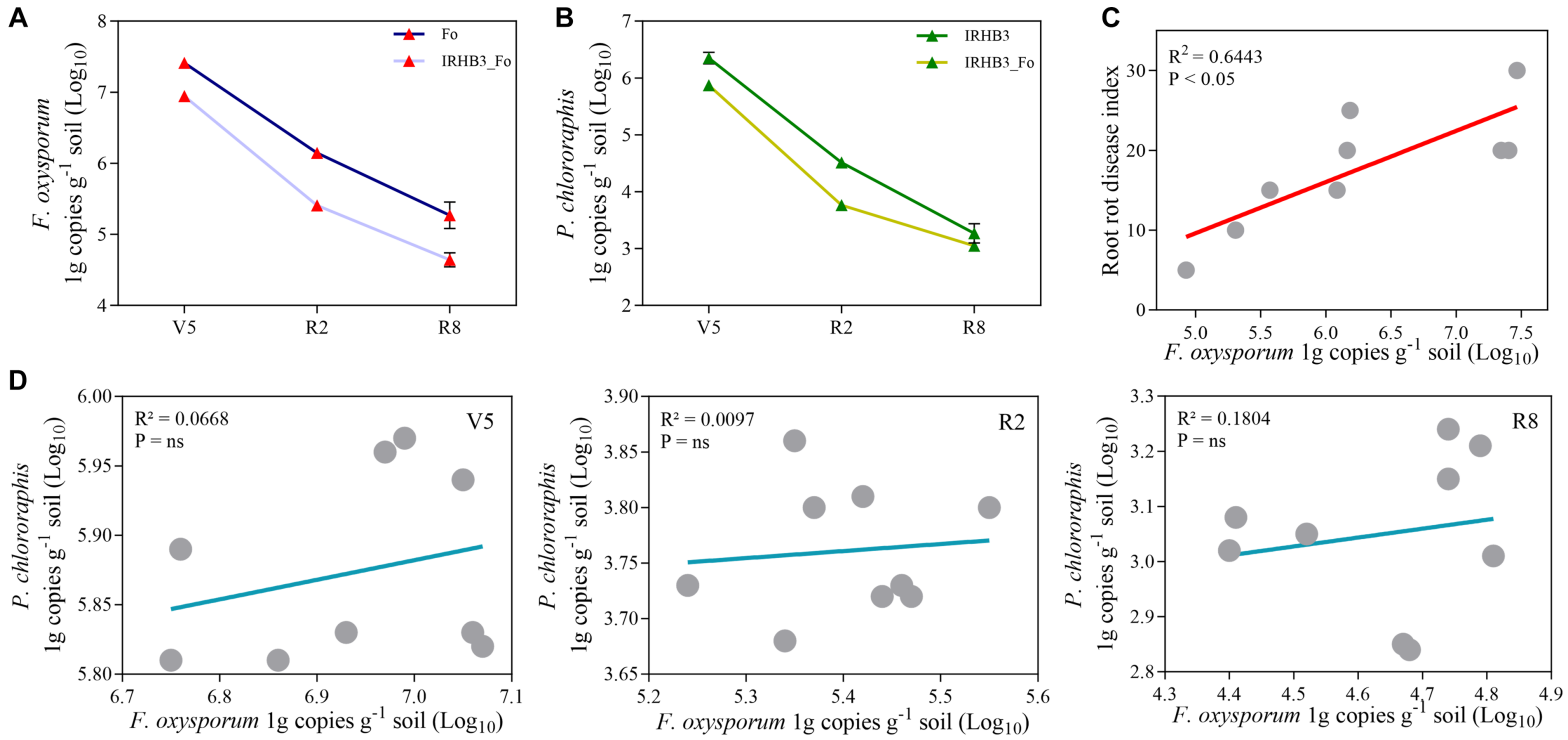
Survival dynamic tracking and correlation analysis of beneficial IRHB3 and pathogen *F. oxysporum* in rhizosphere soils. **(A)** *F. oxysporum* and **(B)** *P. chlororaphis* copies in soybean rhizosphere soil. **(C)** Correlation analysis of *F. oxysporum* abundance and disease index of soybean root rot. **(D)** Correlation analysis of the abundance of *F. oxysporum* and *P. chlororaphis* IRHB3 at V5, R2, and R8 periods. R2 represents the correlation coefficient. *p* < 0.05 and *p* = ns indicate a significant difference and no difference, respectively.

Since most exogenously applied microorganisms are relatively difficult to survive and colonize in the complicated field environment, their work amounts generally need to account for 10^5^ cfu·g^−1^ (Pieterse et al., 2014; Liu et al., 2021). In this study, copies of *gyrB* and *ITS* fragments were examined by qRT-PCR to record the survival abundance and dynamics of *P. chlororaphis* IRHB3 and *F. oxysporum* B3S1 in soybean rhizosphere soil. As shown in Figure 2, both *P. chlororaphis* and *F. oxysporum* copies in rhizosphere soil gradually decreased from V5 to R8 stages, but the overall survival ability remained relatively strong. Specifically, *F. oxysporum* gene copies were recorded as 10^7^, 10^6^, and 10^5^ cfu·g^−1^ and 10^6^, 10^5^, and 10^4^ cfu·g^−1^ at V5, R2, and R8 stages of soybean growth in the treatments of Fo and IRHB3_Fo, respectively (Figure 2A), whereas *P. chlororaphis* copies accounted for 10^6^, 10^4^, and 10^3^ cfu·g^−1^ and 10^5^, 10^3^, 10^3^ cfu·g^−1^ in the corresponding treatments of IRHB3 and IRHB3_Fo at three stages in sequence (Figure 2B). There was a significant positive correlation between the disease index of root rot and *F. oxysporum* copies (*R*^2^ = 0.6443, *p* < 0.05) (Figure 2C), indicating that the more abundant *F. oxysporum*, the more serious root rot disease. Furthermore, the correlation coefficients R2 of *P. chlororaphis* and *F. oxysporum* copies were 0.0668, 0.0097, and 0.1804 at the V5, R2, and R8 stages, respectively (Figure 2D), demonstrating that there was no close correlation (P = ns) between *P. chlororaphis* and *F. oxysporum*.

Although the abundance of *F. oxysporum* gradually decreased with soybean growth, its concentration remained as high as 10^5^ cfu·g^−1^ in *F. oxysporum*-treated rhizosphere soil, which is still enough to exert pathogenic functions. In addition, *F. oxysporum* abundance in IRHB3_Fo treatment reached the working concentration in both V5 (10^6^ cfu·g^−1^) and R2 (10^5^ cfu·g^−1^) periods, but the occurrence of root rot disease was never observed. For the biocontrol bacterium, IRHB3 only reached the working concentration (10^6^ cfu·g^−1^ in IRHB3 treatment, 10^5^ cfu·g^−1^ in IRHB3_Fo treatment) at V5 period, and after that, it was always lower than 10^5^ cfu·g^−1^, indicating that IRHB3 might not individually service host to confront pathogen attack.

### 3.4 IRHB3 increases soybean rhizosphere bacterial diversity but does not alter the structure

**Figure 3:**
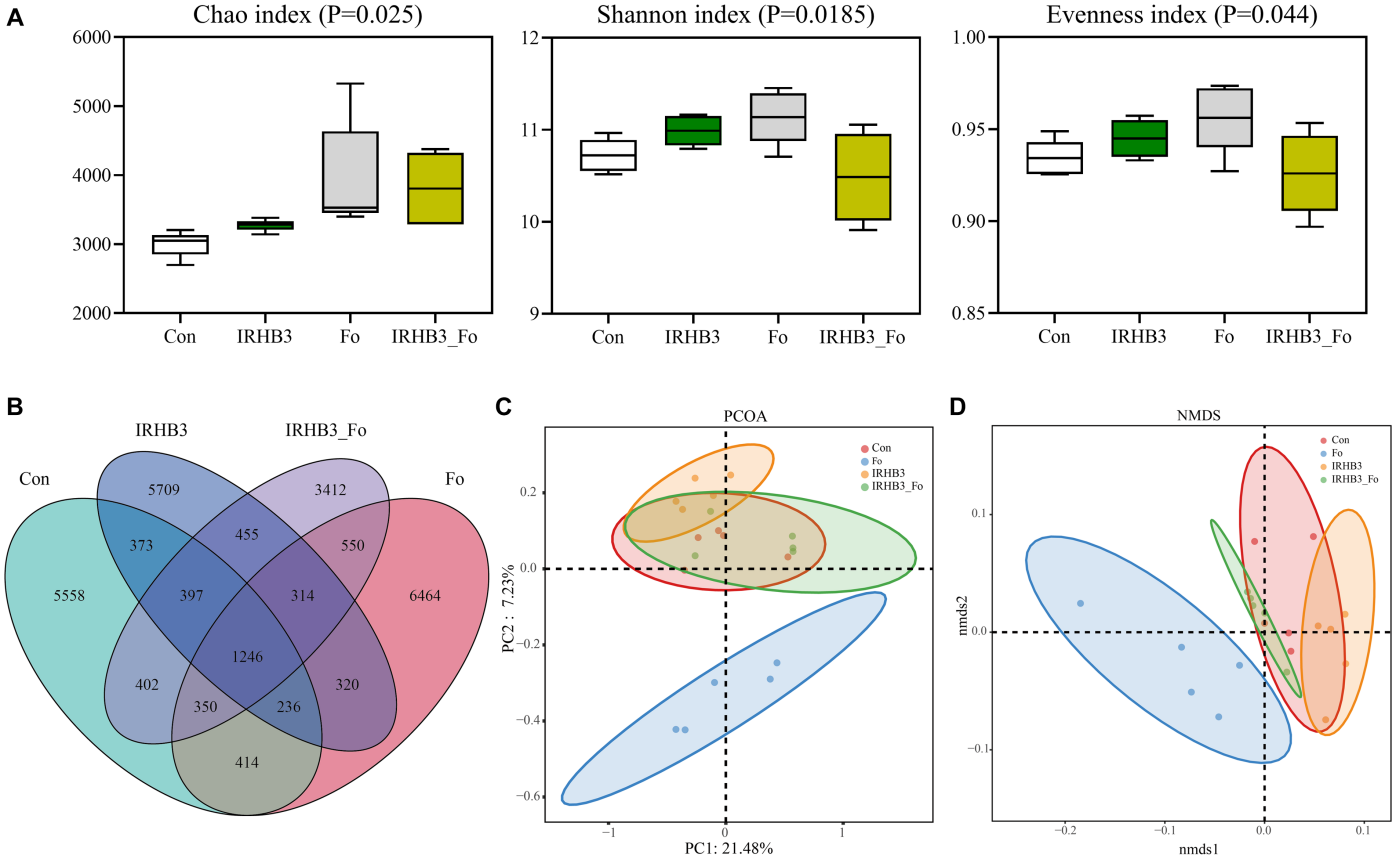
The diversity, structure, and abundance of the rhizosphere bacteria were community affected by four different treatments. **(A)** Alpha diversity analysis. **(B)** Venn diagram of rhizosphere bacterial OUTs. **(C)** PCOA principal component analysis. **(D)** NMDS structure analysis.

To investigate the impacts of different treatments on the local bacterial community in the field, the diversity and structure of the rhizosphere bacterial community were analyzed and compared through *16S rRNA* high-throughput sequencing. Alpha diversity analysis showed that IRHB3 treatment (IRHB3) improved the richness (Chao index), diversity (Shannon index), and evenness (Evenness index) of soybean root bacteria (*p* < 0.05) when compared with control (Con) (Figure 3A). As expected, *F. oxysporum* inoculation (Fo) also significantly changed the bacterial community abundance, but IRHB3 supplement (IRHB3_Fo) partially returned the bacterial species diversity and evenness. To compare the numbers of OTUs, we found that the numbers of unique bacterial OTUs were 5,558 for Con, 5,709 for IRHB3, 6,464 for Fo, and 3,412 for IRHB3_Fo, respectively. There were the most shared OTUs between IRHB3_Fo and Fo treatments (2460) and the lowest shared OTUs between IRHB3 and Fo treatments (2116) (Figure 3B), which indicates the more similar bacterial community diversity in the corresponding comparison group.

Beta diversity analysis was conducted through PCOA and NMDS analyses based on Weighted UniFrac distance (Figures 3C, D). PCOA analysis showed that except for *F. oxysporum*, the other three treatments had a relatively close relationship and clustered together in the second principal component (PC2) (Figure 3C). NMDS analysis had the same trend as PCOA analysis, and the treatments of IRHB3 and IRHB3_Fo had partial overlap with the healthy control (Con), implying the same structure of the bacterial community. In contrast, the treatment of IRHB3_Fo was totally separated from *F. oxysporum*, indicating the bacterial structure was clearly altered because of the IRHB3 supplement (Figure 3D). Overall, IRHB3 could improve the abundance of the rhizosphere bacterial community but not the structure, especially since this strain displays good impacts on recovering the bacterial diversity and structure affected by *F. oxysporum*.

### 3.5 IRHB3 does not directly recruit beneficial biological microbes in soybean rhizosphere

**Figure 4:**
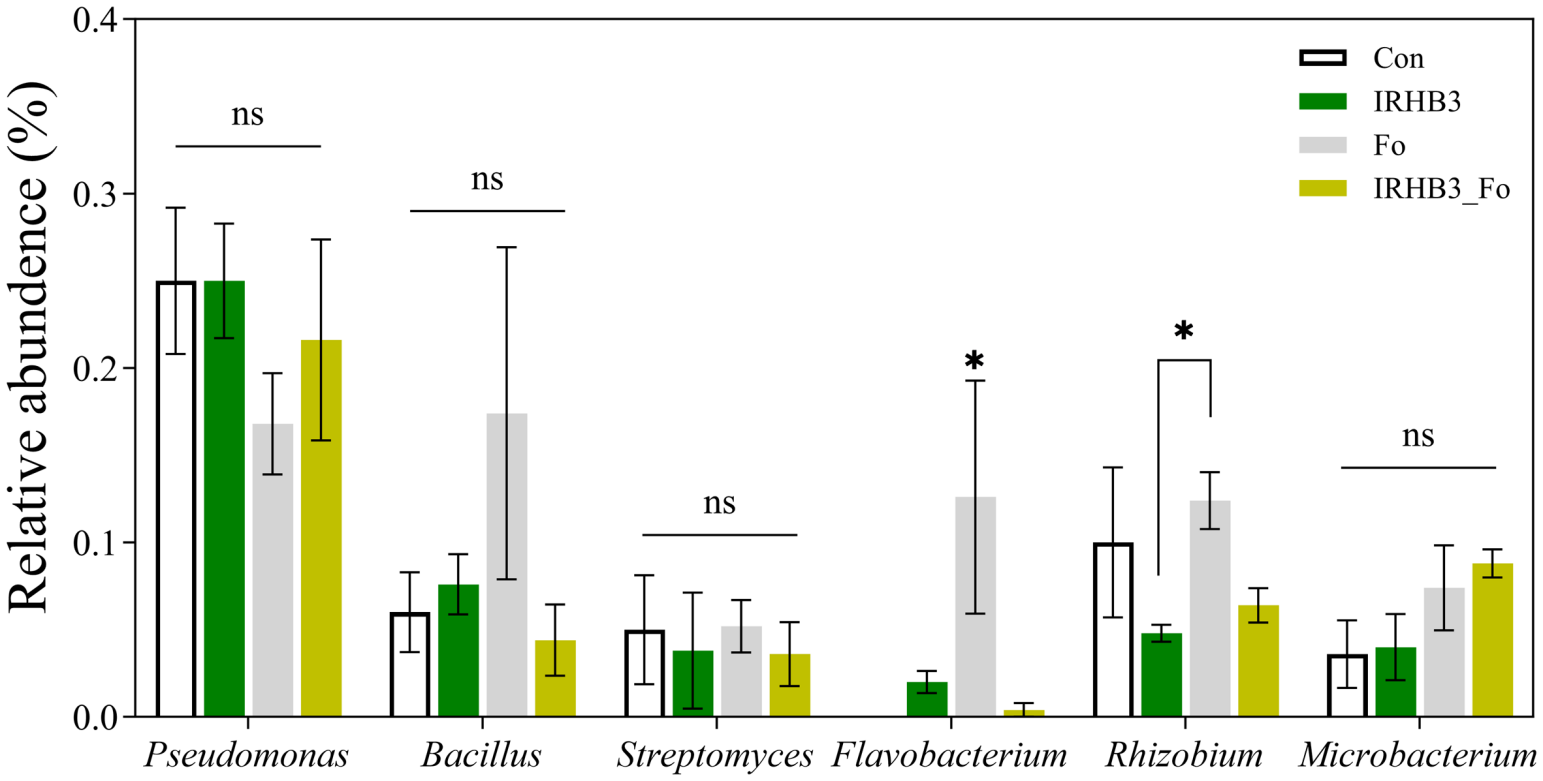
Relative abundance of six species of beneficial bacteria. ns represents no significant difference, whereas asterisks indicate significant differences (*p* < 0.05) according to Duncan’s multiple range test.

Previous studies demonstrated that *Pseudomonas*, *Bacillus*, *Streptomyces*, *Flavobacterium*, *Rhizobia*, and *Microbacterium* were common beneficial microorganisms in the soil. Therefore, we compared the relative abundance of these bacterial genera in rhizosphere soil from four treatments. As shown in Figure 4, there were no significant differences in the abundance of rhizosphere *Pseudomonas*, *Bacillus*, *Streptomyces*, and *Microbacterium* among the four treatments. In contrast, *Flavobacterium* was considerably recruited by both *F. oxysporum* and IRHB3 as compared to the control, whereas *Rhizobium* was preferably accumulated by *F. oxysporum* rather than IRHB3. These results demonstrate that neither IRHB3 nor *F. oxysprum* have any direct impacts on *Pseudomonas*, *Bacillus*, *Streptomyces*, or *Microbacterium*, but they might have certain competition on the activation or re-activation of *Flavobacterium* and *Rhizobium*.

### 3.6 IRHB3 recruits functional bacteria related to phosphorus and nitrogen utilization for rhizosphere colonization

**Figure 5:**
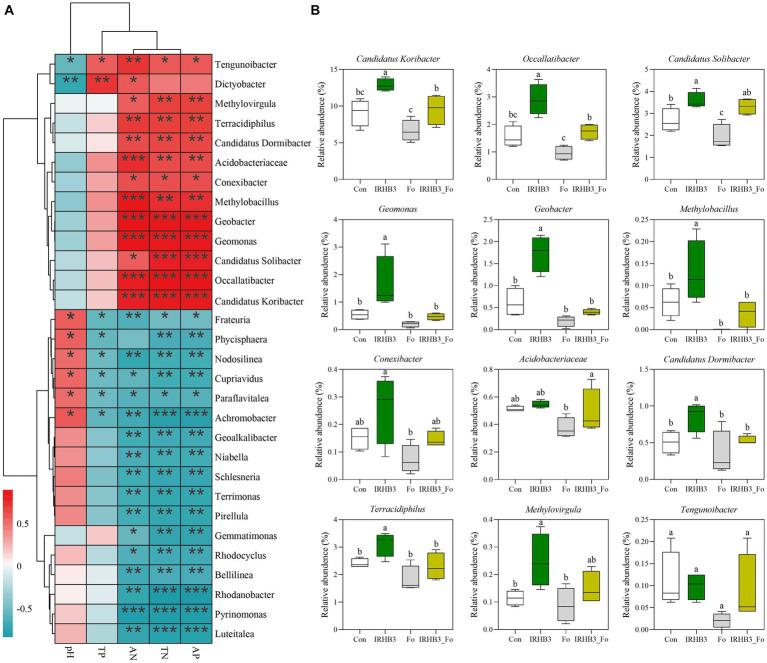
Function-based bacterial analysis related to phosphorus solution and nitrogen fixation among different treatments. **(A)** Correlation analysis of soil chemistry and bacterial communities. Red represents a positive correlation; blue represents a negative correlation. *p < 0.05; **p < 0.01; ***p < 0.001. **(B)** Relative abundance of phosphorus solution and nitrogen fixation functional bacteria.

Nitrogen and phosphorus, as essential nutrients for plant growth and development, have an influence on the structure of the rhizosphere microbial community. In return, some soil microbes have the ability to activate or convert unusable nitrogen and phosphorus into usable forms to meet the nutrient demands of plants (Agren et al., 2012). In the current study, 12 bacterial genera, including *Tengunoibacter*, *Dictyobacter*, *Methylovirgula*, *Terracidiphilus*, *Candidatus dormibacter*, *Acidobacteriaceae*, *Conexibacter*, *Methylobacillus*, *Geobacter*, *Geomonas*, *Candidatus solibacter*, *Occallatibacter*, and *Candidatus koribacter*, were positively correlated with soil available phosphorus (AP), alkaline nitrogen (AN), and total nitrogen (TN). In addition, 17 genera of bacteria, like *Frateuria*, *Phycisphaera*, *Nodosilinea*, *Cupriavidus*, *Paraflavitalea*, *Achromobacter*, *Geoalkalibacter*, *Niabella*, *Schlesneria*, *Terrimonas*, *Pirellula*, *Gemmatimonas*, *Rhodocyclus*, *Bellilinea*, *Rhodanobacter*, *Pyrinomonas*, and *Luteitalea*, were negatively correlated with AP, AN, and TN, implying their negative effects on the nutrient uptake of soybean (Figure 5A).

To further uncover whether these functional bacteria related to nitrogen and phosphorate uptake and utilization can be affected by the biocontrol strain IRHB3 or the pathogen *F. oxysporum*, the relative abundance of these genera in rhizosphere soil was compared among four different treatments. As shown in Figure 5B, except for *Conexibacter*, *Acidobacteriaceae*, and *Tengunoibacter*, IRHB3 effectively increased the relative abundance of 10 potential phosphorus-solubilizing and nitrogen-fixing function bacteria in the soybean rhizosphere as compared to the control. *F. oxysporum* inoculation had almost no impact on the relative abundance of these functional bacteria; however, once IRHB3 was added to the *F. oxysporum*-inoculated soil, it largely decreased the abundance of seven functional bacteria, including *Candidatus Koribacter*, *Occallatibacter*, *Geomonas*, *Geobacter*, *Methylobacillus*, *Candidatus Dormibacter*, and *Terracidiphilus*, which obviously were increased by IRHB3, indicating that *F. oxysporum* abolished the positive effects of IRHB3 on phosphorus and nitrogen utilization (Figure 5B).

**Figure 6:**
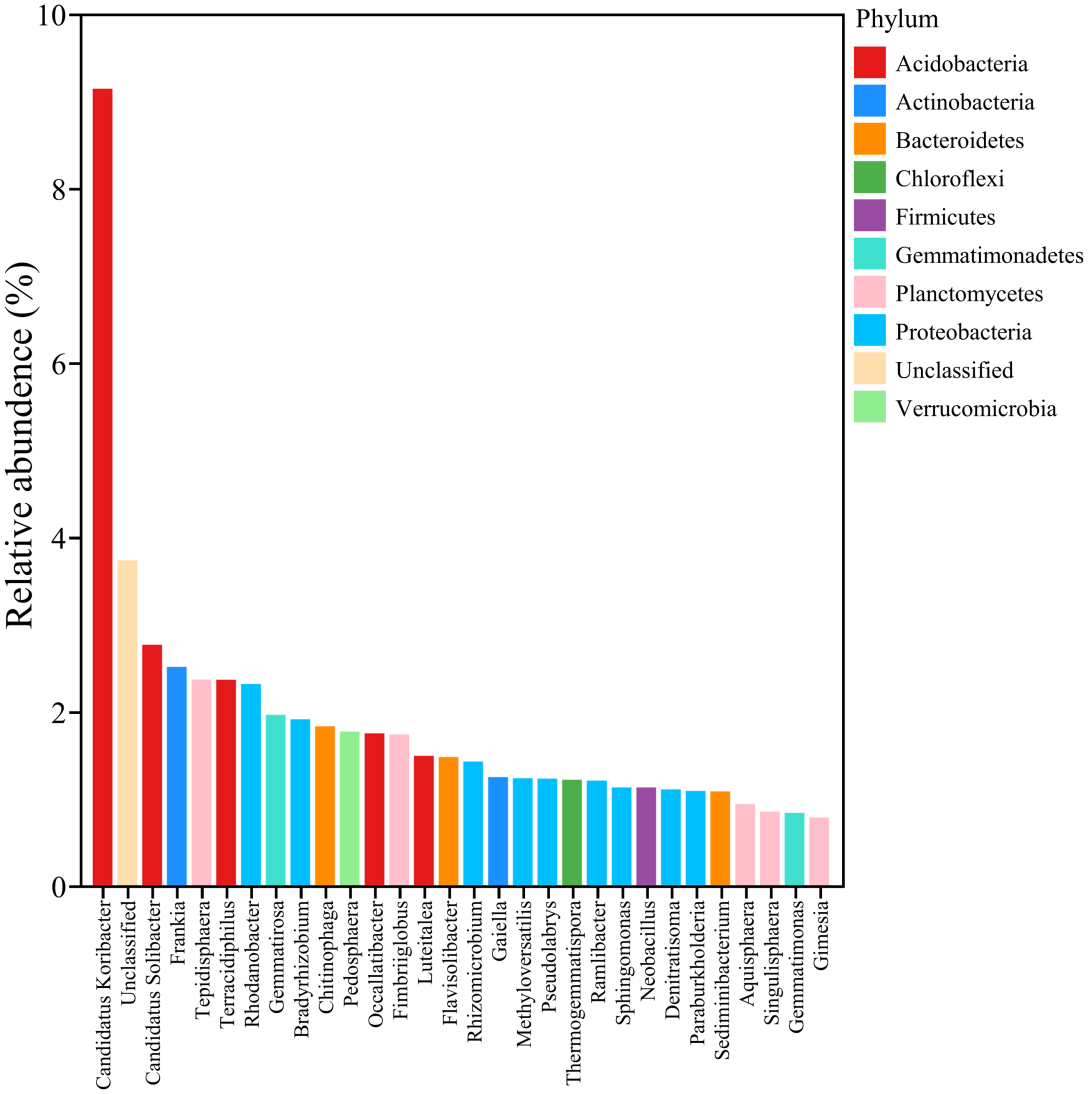
Core bacteria in rhizosphere soil (top 30).

Moreover, the annotation data based on high-throughput sequencing showed that five functional bacteria, such as *Geobacter*, *Geomonas*, *Candidatus solibacter*, *Occallatibacter*, and *Candidatus koribacter*, belonged to core bacteria in the rhizosphere soil of soybean, as shown in Figure 6, and meanwhile, compared with the control, IRHB3 administration increased the relative abundance of these five functional bacteria. Compared with *F. oxysporum*, the addition of IRHB3 to *F. oxysporum*-treated soil recovered the relative abundance of *Candidatus koribacter*, *Occallatibacter*, *Candidatus solibacter*, and *Terracidiphilus* to some extent. Furthermore, *Methylobacillus* was the unique functional bacterium recruited by IRHB3 but was not detected in *F. oxysporum*-inoculated treatment.

### 3.7 IRHB3 depends on the JA signaling pathway to induce systemic resistance

**Figure 7:**
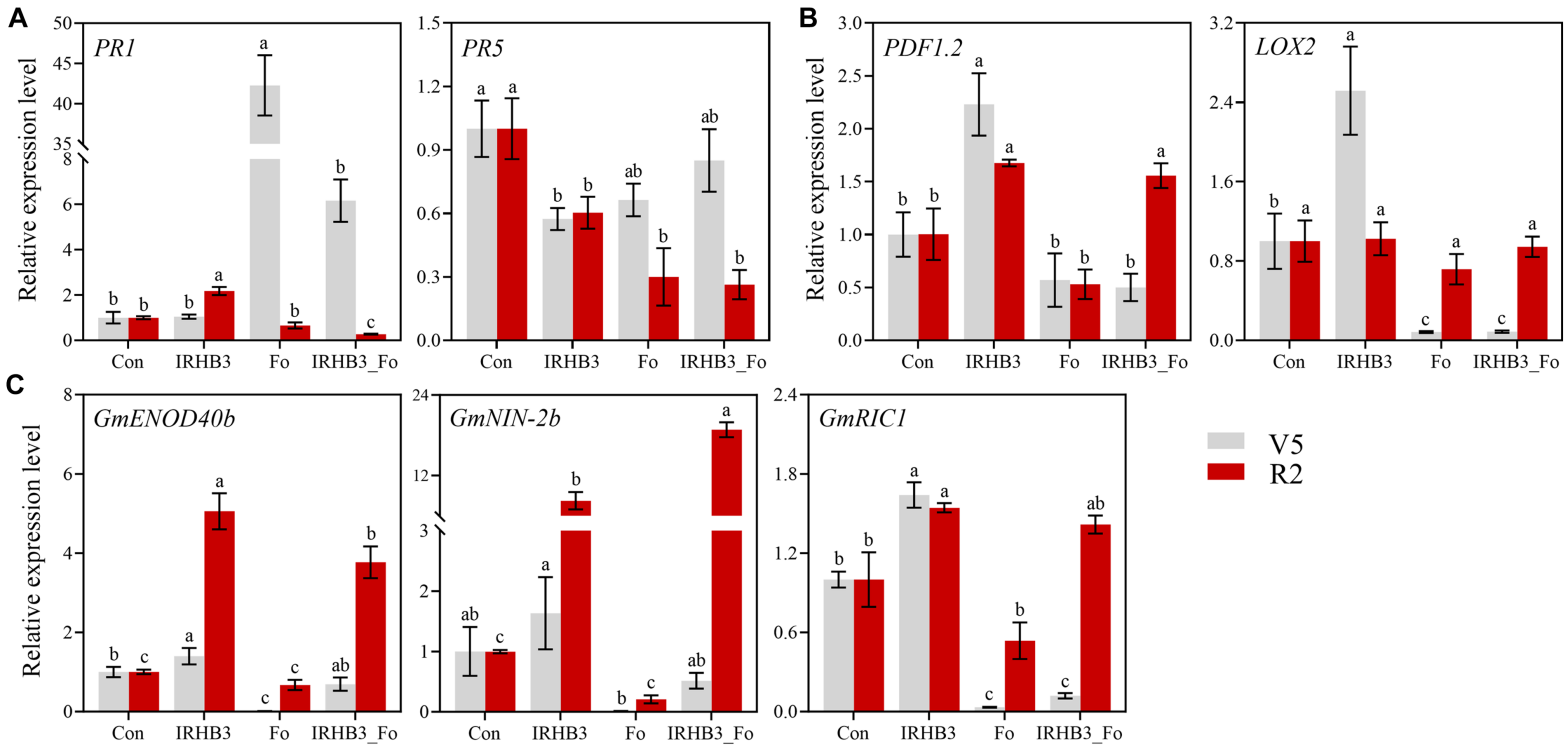
Expression analysis of defense and nodulation signaling marker genes in soybean root. **(A)** Relative expression levels of *PR1* and *PR5* related to SA signaling. **(B)** Relative expression levels of *PDF1.2* and *LOX2* related to JA signaling. **(C)** Relative expression levels of *GmENOD40b*, *GmNIN-2b*, and *GmRIC1* related to nodulation signaling Gene relative expression was calculated using the 2^−ΔΔ Ct^ method with the *GmActin* gene as a reference for normalization. The gray and red bars represent the relative expression levels of marker genes at the V5 and R2 periods, respectively. Different lowercase letters in the same color bar indicate significant differences (*p* < 0.05) according to Duncan’s multiple range test.

To find out whether IRHB3 was able to induce host resistance, both *PR1* and *PR5* were selected as the markers for SA signaling-mediated SAR, whereas *PDF1.2* and *LOX2* were used as the detection for JA signaling-mediated ISR. Relative gene expression was examined from soybean roots at V5 and R2 growth stages from four different treatments. As shown in Figure 7A, compared with the control, *PR1* was remarkably induced by *F. oxysporum* at the V5 stage, but it decreased after IRHB3 addition. Although IRHB3 had no significant effect on *PR1* expression, it significantly reduced *PR1* expression levels induced by *F. oxysporum* (Figure 7A). At the R2 stage, *F. oxysporum* did not alter *PR1* expression as compared to the control, but the IRHB3 supplement induced a lower expression level of *PR1* as compared to *F. oxysporum*. For the *PR5* gene, there were similar trends of gene expression activation at the V5 and R2 stages. Interestingly, all three treatments of IRHB3, *F. oxysporum*, and IRHB3_Fo significantly decreased *PR5* expression as compared to the control, when IRHB3 addition had almost no impact on *PR5* expression, which was downregulated by *F. oxysporum*. Therefore, neither IRHB3 nor *F. oxysporum* could induce *PR5*-dependent soybean resistance. Thus, IRHB3 and *F. oxysporum* have distinct impacts on *PR1* and *PR5* expression. *F. oxysporum* inoculation trends to activate *PR1*-mediated SA signaling, whereas IRHB3-triggered resistance might not or at least not totally depend upon this pathway.

JA signaling is often activated by wounding or necrotrophic pathogen attacks and is often related to induced systemic resistance (ISR). In this study, as shown in Figure 7B, we found that IRHB3 dramatically induced *PDF1.2* and *LOX2* gene expression as compared to the control at the V5 growth stage of soybean, but IRHB3 addition to the soil did not affect the expression of both *PDF1.2* and *LOX2*, which were not triggered by *F. oxysporum* inoculation. At the R2 stage, although IRHB3 continuously triggered *PDF1.2* expression and even enhanced *PDF1.2* expression when co-treated with *F. oxysporum*, it failed to activate *LOX2* expression. In general, IRHB3 prefers to activate JA-mediated ISR rather than SA-dependent SAR to confront pathogen attacks in the soybean rhizosphere.

### 3.8 IRHB3 induces expression of nodulation genes to promote symbiotic nitrogen fixation

As shown in Figure 7C, IRHB3 observably increased the expression level of nodulation-related genes *GmENOD40b* and *GmRIC1* of soybean roots rather than control, but it had almost no statistically significant influences on *GmNIN-2b* expression at V5 stages. In comparison, *F. oxysporum* remarkably suppressed the expression of *GmENOD40b* and *GmRIC1*, but IRHB3 addition into *F. oxysporum*-infected soil (IRHB3_Fo) effectively re-activated *GmENOD40b* and also weakly affected the other two genes. Surprisingly, IRHB3 induced much higher expression of *GmENOD40b*, *GmNIN-2b*, and *GmNIN-2b* at the R2 stage as compared to control, and meanwhile, even IRHB3 co-inoculation with *F. oxysproum* (IRHB3_Fo) did not decrease the high expression level of three genes. In particular, *GmNIN-2b* had an even higher level than that in IRHB3-treated roots. Thus, IRHB3 could activate nodulation gene expression to promote root nodule formation, which is rarely affected by pathogen attacks.

## 4 Discussion

The rhizosphere is the core area of the plant–microbe–pathogen interaction that determines the occurrence of soil-borne diseases (Raaijmakers et al., 2008). The rhizosphere microbiome has recently been recognized as the first defense line to limit pathogen invasion and maintain plant health (Eisenhauer et al., 2010; Berendsen et al., 2012). In particular, beneficial rhizosphere microbes play an important role in plant health and defenses, and some of them have also been widely applied as biocontrol agents for soil-borne disease control. IRHB3 was screened from the intercropped soybean rhizosphere, and it not only displayed good antagonistic effects on the main *Fusarium* species of soybean root rot as well as several agricultural fungi but also exhibited certain growth promotion (Chang et al., 2022; Wei et al., 2023). Here, we further find out the disease biocontrol and growth-promoting mechanisms of *P. chlororaphis* IRHB3 when introduced into field soil. We found that IRHB3 significantly improved root development and increased root nodules. In particular, when supplemented with *F. oxysporum*-inoculated soil, IRHB3 nearly suppressed root rot, alleviated the negative effects caused by *F. oxysporum*, and rescued the root nodulation ability. These results are consistent with those previously examined in the greenhouse (Wei et al., 2023).

The diversity and structure of the rhizosphere microbiome play an important role in the homeostasis of rhizosphere microecology (Eisenhauer et al., 2010). A higher microbial diversity and suitable combination are more conducive to hosts when coped with environmental stimuli (Garbeva et al., 2004; Wei et al., 2015). Our previous studies found that the rhizosphere microorganisms of strip intercropped soybeans had a higher diversity and attracted more beneficial microorganisms as compared to those under soybean monoculture, ultimately resulting in stronger resistance to root rot (Chang et al., 2022; Xu et al., 2022). In the current study, IRHB3 application improved the diversity of the rhizosphere bacterial community, and IRHB3 also had remarkable advantages in recovering the rhizosphere bacterial diversity that was altered by the pathogen *F. oxysporum*, thus maintaining the homeostasis of the healthy rhizosphere microbiome in plant hosts.

In addition, Xiong et al. (2017) and Tao et al. (2020) found that the application of bio-fertilizer *Bacillus* in field soil recruited more PGPR *Pseudomonas* sp., *Bacillus* sp., etc. for colonization in the host rhizosphere. Chihaoui et al. (2015) reported that inoculation of nodule-endophyte *Agrobacterium* sp. 10C2 altered the abundance and structure of rhizosphere bacteria and increased the relative abundance of PGPR *B. licheniformis*, *B. pumilus*, *B. senegalis*, etc., thereby promoting root nodulation and growth. Previous studies have shown that maize–soybean strip intercropping is capable of recruiting much richer and more diverse species of *Pseudomonas*, *Bacillus*, *Streptomyces*, and *Microbacterium* in the soybean rhizosphere when compared with monoculture (Chang et al., 2022). However, as one representative beneficial bacterial strain, IRHB3 addition in field soil did not significantly recruit *Pseudomonas*, *Bacillus*, *Streptomyces*, and *Microbacterium* for colonization in the soybean rhizosphere as compared to *F. oxysporum* and the blank control. In contrast, the pathogen *F. oxysporum* inoculation attracted some beneficial genera like *Flavobacterium* and *Rhizobium*. This can be explained by the “Cry help” of host plants, which tends to recruit beneficial microbiome colonization to build an alliance to resist pathogen infection (Liu et al., 2021). Similarly, Yin et al. (2021) found that successive monocultures and pathogen *Rhizoctonia solani* AG8 infection could stimulate wheat to regulate rhizosphere microbiome structure and specifically accumulate a group of beneficial microbiomes, such as *Chitinophaga*, *Pseudomonas*, *Chryseobacterium*, *Flavobacterium*, and *Rhizobium*, to inhibit fungal pathogens. Wen et al. (2020) study showed that the host root could actively secrete long-chain fatty acids, amino acids, etc. to recruit specific beneficial bacteria, *Pseudomonas* sp., to help the host alleviate the damage caused by shoot pathogen infection. Thus, IRHB3 suppression of root rot might not depend on its attraction to other biocontrol bacteria.

Nitrogen (N) and phosphorus (P) are the essential macronutrients for plant growth and development (Pradhan et al., 2015). Unfortunately, most of the N and P in the soil cannot be directly utilized by plants due to their mineralization (Agren et al., 2012; Alori et al., 2017). However, some microbes related to P solubilization and N fixation can effectively improve the uptake and utilization of soil nutrients (Rodríguez and Fraga, 1999). Song et al. (2022) reported nine species of P-solubilizing bacteria (PSBs) from the rhizosphere soil of intercropped soybean, and these functional bacteria increased the solubilization of unavailable P and improved soil P availability through secreting organic acids to promote maize seed germination and seedling growth. Zhang et al. (2022) showed that *Curtobacterium citreum* A02, as a nitrogen-fixing bacterium, promoted cassava growth through nitrogen fixation, P dissolution, and IAA secretion. In this study, we found that IRHB addition increased the contents of available N and P nutrients in soybean rhizosphere soil, and the same effects had also been observed in the co-treatment of IRHB3 and *F. oxysporum*. Analysis of the rhizosphere microbiome demonstrated that a total of 12 bacterial genera were closely related to P solubilization and N fixation, and among them, five genera, including *Geobacter*, *Geomonas*, *Candidatus solibacter*, *Occallatibacter*, and *Candidatus Koribacter,* were the most abundant and core bacteria in field soil. Interestingly, both IRHB3 and IRHB3_Fo treatments could significantly attract those P solubilization and N fixation bacteria for colonization in the soybean rhizosphere. Furthermore, since *GmENOD40b*, *GmNIN-2b*, and *GmRIC1* are early nodulation genes in soybean (Hayashi et al., 2012; Lin et al., 2012), we found that IRHB3 activated expression of these three nodulation genes in the early stages (V5), whereas *F. oxysporum* significantly inhibited the expression of these genes. This supports that IRHB3 promotes root development and increases root nodules rather than *F. oxysporum*, which is also consistent with previous research (Tyler, 2007; Lin et al., 2012). Based on these results, we can predict that IRHB3 cooperates with local functional bacteria responsible for nutrient availability to improve root development and nodule formation, eventually contributing to growth promotion.

When faced with the complex composition of a field ecosystem, most externally applied beneficial microbes struggle to survive because of poor competition, low colonization, weak environmental adaptation, and other factors (Jez et al., 2016; Zhang et al., 2020). Some researchers pointed out that the abundance of single-species microorganisms in soil needs to account for 10^5^ cfu·g^−1^ at least to perform individual function (Pieterse et al., 2014; Liu et al., 2021). In the current study, the abundance of IRHB3 and *F. oxsporum* was gradually decreased in soybean rhizosphere soil during the growth period through monitoring relative gene copies using RT-qPCR. The amounts of the pathogen *F. oxyporum* were more than 10^5^ cfu·g^−1^ in the whole life of soybean growth to continuously infect soybean, and it had a close correlation with the disease index of root rot. In contrast, IRHB3 in the soybean rhizosphere only maintained a high abundance at the V5 stage, and after that, IRHB3 still survived, but its overall population was probably not enough to provide specific help for the host in an individual way. Moreover, correlation analysis shows that there was no significant correlation between *P. chlororaphis* and *F. oxysporum* in soil. This indicates that IRHB3 does not directly defend against *F. oxysporum* to suppress root rot. Furthermore, beneficial microbes often induce host resistance to cope with pathogen invasion. Yang et al. (2023) reported that the application of *B. proteolyticus* OSUB18 activated ISR through upregulating expression levels of genes *PDF1.2*, *LOX3*, etc. In this study, although IRHB3 did not significantly recruit a set of other beneficial biocontrol microbes in the rhizosphere, its addition dramatically activated high-level expression of *PDF1.2* and *LOX2*, which are responsible for JA-mediated ISR, rather than *PR1*, which is related to SA-mediated SAR. Thus, IRHB3 might trigger JA-mediated ISR to confront *F. oxysporum* infection.

**Figure 8:**
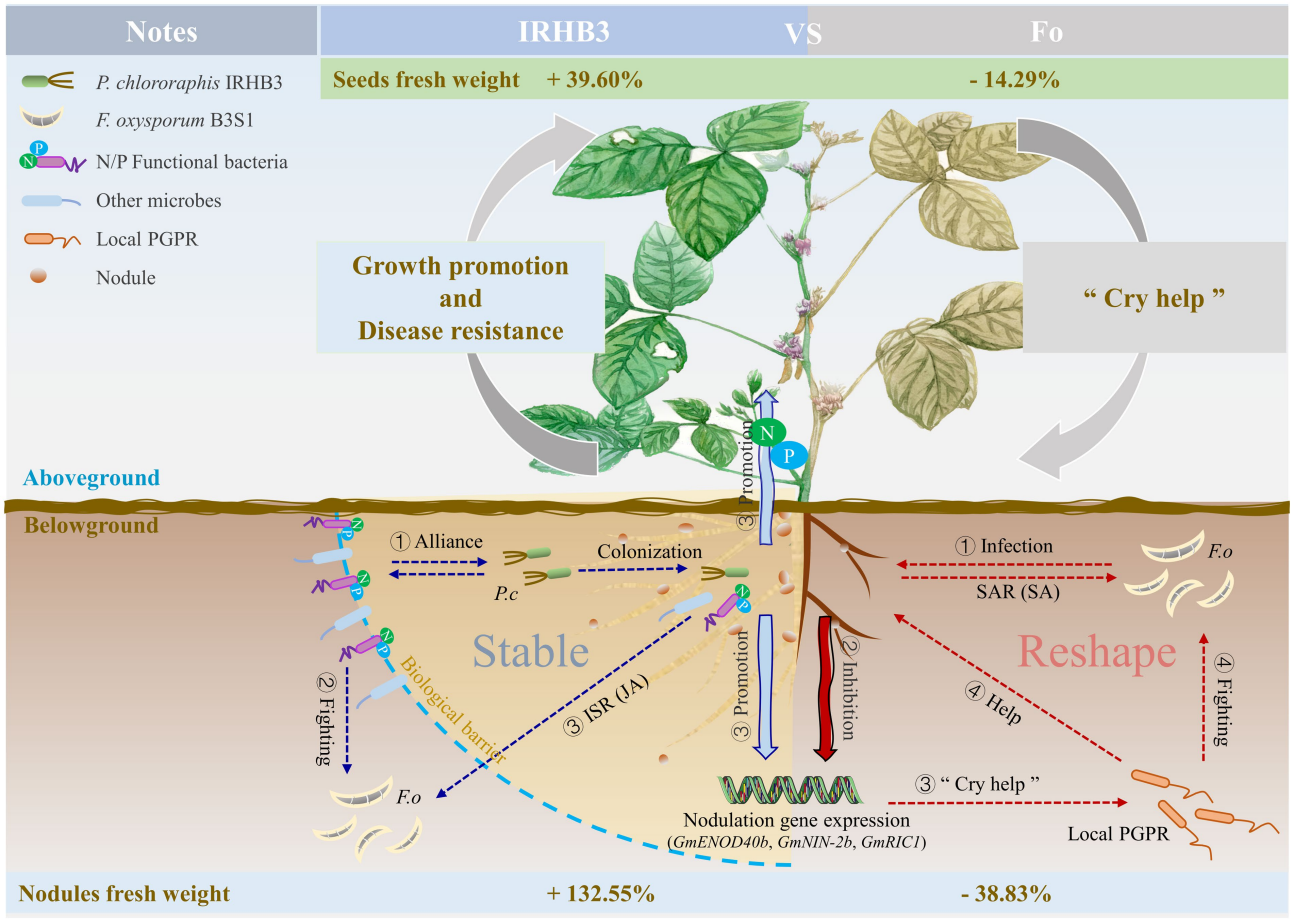
Schematic diagram of the mechanism of soybean growth promotion and disease suppression in the field for IRHB3.

Above all, we depicted one simple work model of IRHB3 when applied to field soil and interacted with the pathogen *F. oxysporum*, as shown in Figure 8. As the pathogen microbe of root rot, *F. oxysporum* inoculation changes the diversity of the rhizosphere microbial community, especially reducing some species related to P and N utilization, inhibiting the expression of nodulation genes, and thus severely affecting plant root development, nodule formation, and soybean yield. On the plant side, to ensure survival, the “Cry help” strategy in soybeans is rapidly activated. Typically, a high abundance of beneficial microbes like *Flavobacterium* are rapidly recruited in the soybean rhizosphere, and plant SAR is activated to fight with or suppress pathogen infection while plant-promoting microbes like *Rhizobium* partially maintain plant growth. In contrast, *P. chlororaphis* IRHB3 is one typical PGPR beneficial to soybeans. When added to field soil, it significantly optimizes the diversity of the soybean rhizosphere microbiome, recruits more microbes responsible for P and N utilization, and activates a set of nodulation gene expression that contribute to root development and the increase in root nodules and soybean yield. In addition, JA-mediated ISR can also be induced to enhance plant adaptation to external stimuli. Due to the IRHB3 biocontrol function, the application of IRHB3 into *F. oxysporum*-inoculated soil effectively improves root development and nodulation ability and alleviates the symptoms of root rot.

## 5 Conclusion

This study demonstrates that the application of IRHB3 in the field could increase bacteria diversity and recruit more P solubilization and N fixation functional bacteria for colonization in the soybean rhizosphere. IRHB3 also enhanced host symbiotic nitrogen fixation efficiency by activating nodule-related gene expression to promote plant growth. When plants were faced with rhizosphere microstructure changes caused by the pathogen *F. oxysporum*, IRHB3 could effectively alleviate this stress by maintaining the healthy homeostasis of rhizosphere bacteria. Moreover, IRHB3 and their microbiome could trigger ISR, depending on the JA signaling pathway, to improve host disease resistance. Due to the growth promotion and disease resistance characteristics of IRHB3 in collaboration with the local microbiome, the soybean seed harvest was significantly increased upon IRHB3 treatment and even when combined with *F. oxysporum* inoculation.

## Data availability statement

The original contributions presented in the study are included in the article/Supplementary material, further inquiries can be directed to the corresponding author.

## Author contributions

DW: Data curation, Methodology, Software, Visualization, Writing – original draft, Validation, Writing – review & editing. DZ: Validation, Visualization, Writing – original draft. YZ: Data curation, Writing – original draft, Software. ZY: Software, Writing – original draft. YH: Software, Writing – original draft. CS: Funding acquisition, Writing – review & editing. WY: Funding acquisition, Writing – review & editing. XC: Funding acquisition, Writing – review & editing, Project administration.

## Footnotes

^1^ http://drive5.com/uparse/

^2^ http://rdp.cme.msu.edu/

^3^ http://www.cytoscape.org

^4^ http://bioinformatics.psb.ugent.be/webtools/Venn
