# Supplementary material for: Pseudomonas chlororaphis IRHB3 assemblies beneficial microbes and activates JA-mediated resistance to promote nutrient utilization and inhibit pathogen attack
Source: Front Microbiol. 2024 Feb 5;15:1328863. doi: 10.3389/fmicb.2024.1328863 (PMC10877055; doi:10.3389/fmicb.2024.1328863)
Supplement: Supplementary file 1 [file Data_Sheet_1.docx]

Supplementary Material

# Supplementary Tables S1

**Table S1 Primer information used in this study**

| **Gene** | **Primer name** | **Sequence (5’→3’)** | **Annealing temperature (℃)** | **Reference** |
| --- | --- | --- | --- | --- |
| *16S rRNA* | 27F | AGRGTTYGATYMTGGCTCAG | 55 ℃ | Weisburg et al., 1991 |
|  | 1492R | RGYTACCTTGTTACGACTT |  |  |
| *rDNA ITS* | *FOF1* | ACATACCACTTGTTGCCTCG | 58 ℃ | Jiménez-Fernández et al., 2010 |
|  | *FOR1* | CGCCAATCAATTTGAGGAACG |  |  |
| *16S rRNA fragment* | *PC03F* | GCCGACCAGATGGAAATCA | 58 ℃ | -- |
|  | *PC03R* | CAGTTTGATCGGCATGAACAG |  |  |
| *GmActin* | *GmActin-F* | CCATGTTCCCTGGTATTGCTG | 60 ℃ | Tripathi et al., 2023 |
|  | *GmActin-R* | GTATTTTCTCTCCGGTGGTGC |  |  |
| *PR1* | *PR1-F* | ACTGATCCACTCTGGTGGTG | 60 ℃ | Liu et al., 2018 |
|  | *PR1-R* | ACCGAGTTAGCCCAAACGAC |  |  |
| *PR5* | *PR5-F* | ATGGTTTACTTGGCACTGTGCTCTC | 60 ℃ | Liu et al., 2018 |
|  | *PR5-R* | TTACTGGTGGGCGGTACTAGCAGGC |  |  |
| *PDF1.2* | *PDF1.2-F* | CAATGTAACTTAAGTGCCTAATTATG | 60 ℃ | Liu et al., 2018 |
|  | *PDF1.2-R* | CTTATCAGATCTCAATGGAGAAATC |  |  |
| *LOX2* | *LOX2-F* | CATAGTAGTGTTGGTGGGTTGC | 60 ℃ | Lenis et al., 2010 |
|  | *LOX2-R* | TGTTGAGCCAACTAAGTCGAGA |  |  |
| *GmENOD40b* | *GmENOD40b-F* | GAGTGGCGGAAGCAGATACAC | 60 ℃ | [Hayashi et al., 2012](#_ENREF_26) |
|  | *GmENOD40b-R* | CTACATAGCCATAGAGACCCCAATG |  |  |
| *GmNIN-2b* | *GmNIN-2b-F* | TCTCTCATCCATGATCACCATC | 60 ℃ | [Hayashi et al., 2012](#_ENREF_26) |
|  | *GmNIN-2b-R* | ACTGTCCCAGTTGCAGTAGTGG |  |  |
| *GmRIC1* | *GmRIC1-F* | CAAATGCAACAATGGCTACTCG | 60 ℃ | [Hayashi et al., 2012](#_ENREF_26) |
|  | *GmRIC1-R* | GCCATGGAGATTACTAGCCTGC |  |  |

# Supplementary **Figure S1**


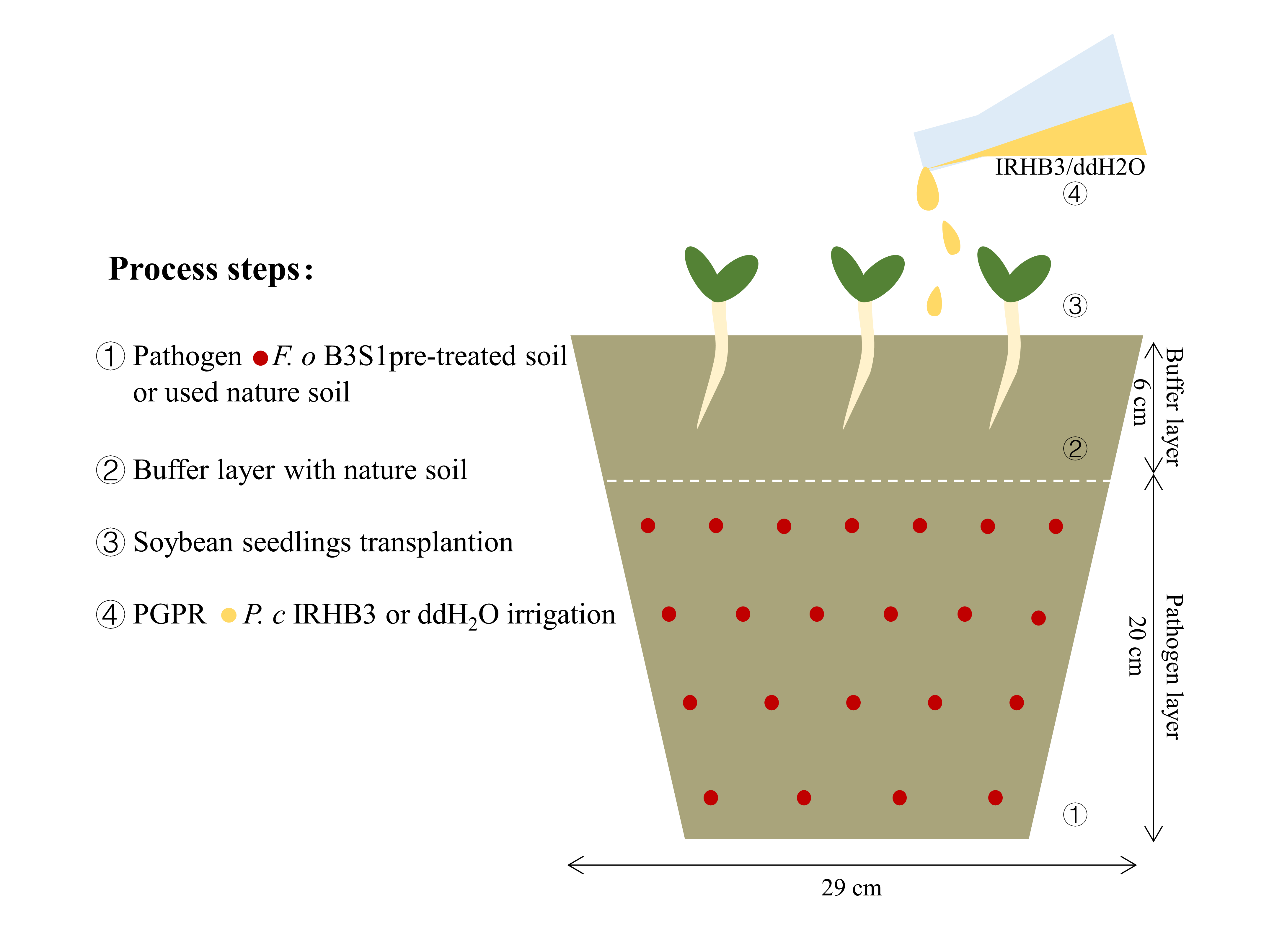


**Figure S1 Schematic diagram of pot experiment treatment**

# Supplementary **Figure S**2


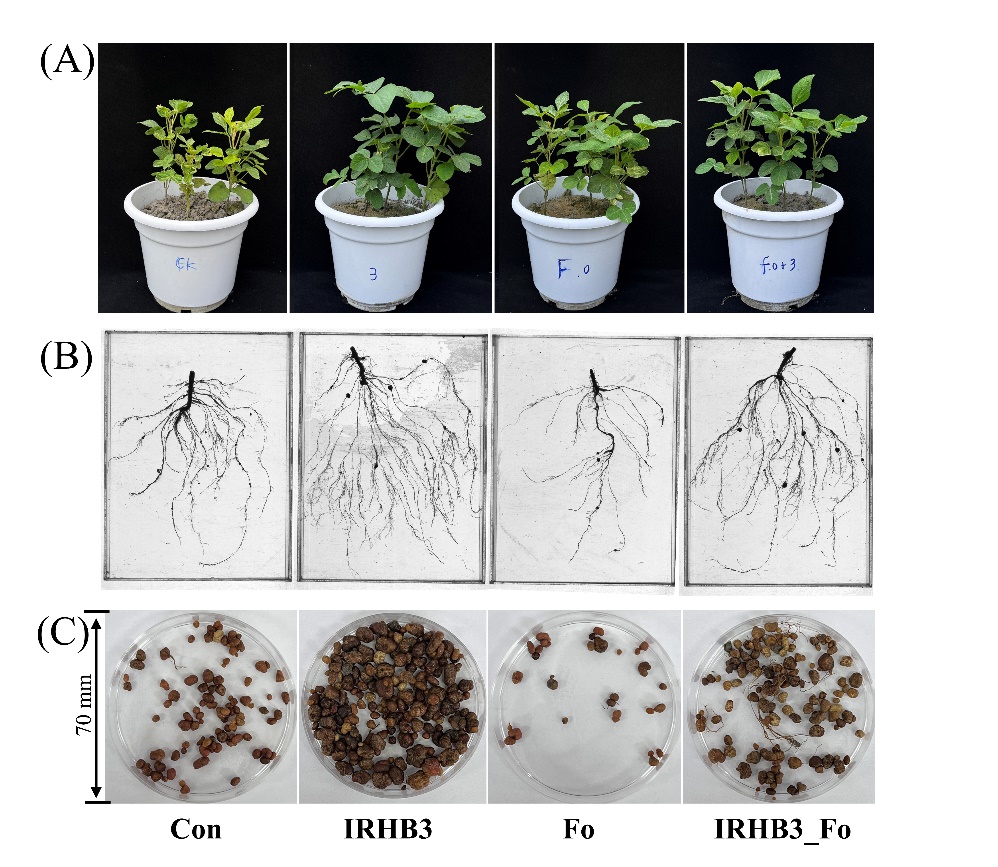


**Figure S2 Effect of IRHB3 on soybean growth at V5 stage.** (A) Shoot growth. (B) Root development. (C) Nodule production.
